# Espial: Electrochemical Soil pH Sensor for In Situ Real-Time Monitoring

**DOI:** 10.3390/mi14122188

**Published:** 2023-11-30

**Authors:** Mohammed A. Eldeeb, Vikram Narayanan Dhamu, Anirban Paul, Sriram Muthukumar, Shalini Prasad

**Affiliations:** 1 Department of Bioengineering, University of Texas at Dallas, Richardson, TX 75080, USA; 2 EnLiSense LLC, Allen, TX 75013, USA; sriramm@enlisense.com

**Keywords:** alizarin, electrochemical sensing, in situ soil pH sensor, real-time continuous soil monitoring, soil texture triangle, squarewave voltammetry

## Abstract

We present a first-of-its-kind electrochemical sensor that demonstrates direct real-time continuous soil pH measurement without any soil pre-treatment. The sensor functionality, performance, and in-soil dynamics have been reported. The sensor coating is a composite matrix of alizarin and Nafion applied by drop casting onto the working electrode. Electrochemical impedance spectroscopy (EIS) and squarewave voltammetry (SWV) studies were conducted to demonstrate the functionality of each method in accurately detecting soil pH. The studies were conducted on three different soil textures (clay, sandy loam, and loamy clay) to cover the range of the soil texture triangle. Squarewave voltammetry showed pH-dependent responses regardless of soil texture (while electrochemical impedance spectroscopy’s pH detection range was limited and dependent on soil texture). The linear models showed a sensitivity range from −50 mV/pH up to −66 mV/pH with R^2^ > 0.97 for the various soil textures in the pH range 3–9. The validation of the sensor showed less than a 10% error rate between the measured pH and reference pH for multiple different soil textures including ones that were not used in the calibration of the sensor. A 7-day in situ soil study showed the capability of the sensor to measure soil pH in a temporally dynamic manner with an error rate of less than 10%. The test was conducted using acidic and alkaline soils with pH values of 5.05 and 8.36, respectively.

## 1. Introduction

**Figure 1:**
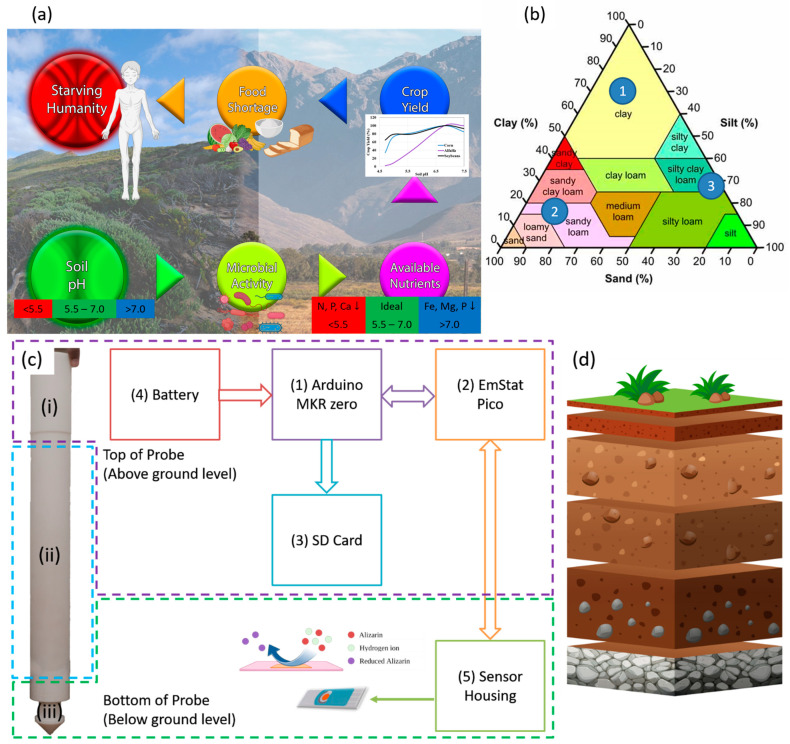
(**a**) How soil pH affects humanity. (**b**) The soil texture triangle provides a breakdown of the different soil types, where the numbers indicate the 3 chosen soil types for sensor calibration. (**c**) Block diagram illustrating the hardware and probe used in this study showing the (i) top, (ii) middle, and (iii) bottom parts. (**d**) A simple breakdown of the change in soil composition at various depths.

Grazing or cropland expansions have resulted in significant deforestation globally [1]. Without adequate monitoring of soil health, once-fertile agricultural land is turning into vast deserts. This does not just affect the food shortage that humanity is facing, but the entire ecosystem. Around 40,000 species are at risk of extinction due to desertification [2]. The United Nations (UN) Sustainable Development Goals (SDG) places sustainable farming as goal 15 [2] while pushing regenerative and sustainable agriculture as a means to combat desertification and deforestation [3]. This requires soil nutrients to be monitored continuously in real time [4,5,6]. Soil pH affects nutrient availability, nutrient leaching, and microorganism activity, in turn affecting plant growth [4,7,8,9,10,11,12]. In turn, this affects crop yield, which results in food shortages if not attended to promptly, leading to global starvation, illustrated by Figure 1a. Different plants require different soil pH ranges. If the soil pH is not properly maintained, fertilizer nutrients would be wasted [10,11,12,13]. Hence, there exists a high demand for low-cost real-time continuous sensing of soil pH.

The current standard for measuring soil pH is not amenable to in situ monitoring. It requires soil sample collection and preparation. The “gold standard” is a potentiometric method that requires mixing the collected soil sample with a balancing reference electrolyte such as potassium chloride and measuring the potential difference. The equilibration conditions are such that it requires over an hour to achieve stability within a lab environment [14]. Due to the need for the reference electrode to be submerged in a saturated KCl solution, it is not feasible to deploy such a glass electrode-based pH sensor in the field. Other methods that overcome this issue include photometric detection [15], conductometric pH sensors based on pH-sensitive hydrogels [16], ion-sensitive field-effect transistor pH sensors [17], remote sensing using satellite images [18], and modified screen-printed electrodes (SPE) [19,20,21]. SPEs are usually modified with quinone compounds, namely polyaniline [21], carbazole-quinone [22], and alizarin [19,20]. Quinone compounds undergo a redox process in the presence of hydrogen ions, where their redox peak potential shifts with changes in hydrogen ion concentration. Reported work in the literature shows a linear relationship between peak potential and pH in the desired soil pH range of 3–9.

However, there has not been a long-term study on the lifetime or reliability of these sensors in situ. Limited studies have been conducted on measuring soil pH using screen-printed electrodes [20] while none have tried to measure soil pH in situ without soil modification. They all require the mixing of soil with a CaCl_2_ solution or KCl buffers prior to measuring. Recently, researchers started looking at electrochemistry to measure various soil parameters [23,24,25]. This work showcases the first-of-its-kind real-time continuous in situ soil pH measurement through the use of alizarin, a quinone that showed the highest accuracy in measuring pH in unbuffered media [19,24]. The study looked at electro-impedance spectroscopy (EIS) and squarewave voltammetry (SWV) as the two electrochemical techniques. Three soil textures were chosen to build the calibration curves in order to cover the soil texture triangle. A 7-day study was conducted to demonstrate the capability of the sensor for continuous monitoring. All results were compared to the gold standard glass electrode. Finally, this work is the first technology proof of feasibility in integrating electrochemical sensors into dynamic soil quality monitoring systems.

## 2. Materials and Methods

### 2.1. Materials

SPE with carbon working and counter electrodes and Ag/AgCl reference electrodes were bought from Metrohm (Herisau, Switzerland). Alizarin, Nafion 117, sodium hydroxide salt, and concentrated hydrochloric acid solution were bought from Sigma Aldrich (Burlington, MA, USA). The hydrochloric acid was diluted to a 1 M concentration using deionized water while 160 mg of sodium hydroxide salt was dissolved in 40 mL of deionized water to prepare the 0.1 M sodium hydroxide solution. A portable potentiostat, the EmStat Pico module, was purchased from PalmSens (Houten, The Netherlands). The computational study was performed using licensed Gaussian software Gaussian 16 W, version 1.1 (Wallingford, CT, USA), Serial # G64254555313819W-2536N. Illustrative sketches were drawn using BioRender (BioRender.com, 11 April 2023).

### 2.2. Soil Sample Preparation

**Figure 2:**
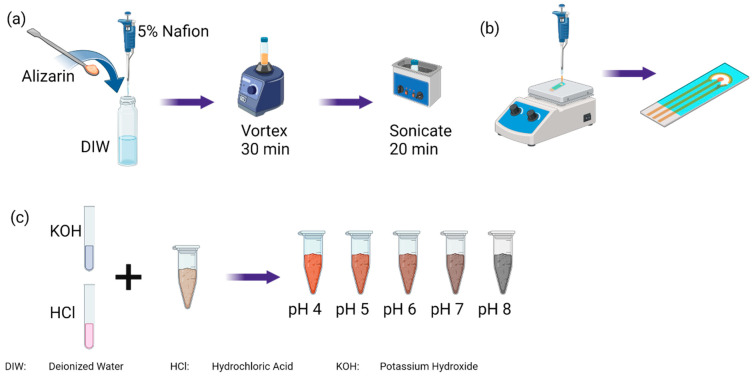
(**a**) Creating a homogenous coating using alizarin and 5% Nafion solution in deionized water followed by (**b**) drop casting of coating at 110 °C onto the working electrode. (**c**) Preparing soil samples with various pH values.

Clay, loamy clay, and sandy loam soils were used in the experiments to build the calibration curves. These soil types were chosen to cover the entire soil texture triangle. The soil was air dried for four days, followed by grinding and filtering through a mesh of 2 mm pores to remove pebbles and procure fine particles of soil. Soil slurries for each of the three soil types were made by mixing 3 g of soil with 1 mL of deionized water. Then, the pH of the soil was adjusted to different values by adding 1 M hydrochloric acid or 0.1 M sodium hydroxide solution in small increments until the desired pH was achieved to cover the range 3–9, as illustrated by Figure 2c. The final water content ranged from 35% to 45%, mimicking agricultural conditions. All soil samples were prepared at least 1 h before the experiment began to ensure the stability of the pH measured.

### 2.3. Electrode Preparation

To prepare the pH-sensitive coating, 5 mg of alizarin was mixed with 950 μL of deionized water, as illustrated in Figure 2a. Then, 50 μL of 5% Nafion 117 was added to the solution and mechanically stirred for 30 min followed by a sonic bath for 20 min. The mixture was then mechanically stirred for 30 s before the preparation of every electrode. An amount of 20 μL was pipetted onto the working electrode at 110 °C drop by drop. The sensor was left on a hot plate at 110 °C for 20 min followed by 2 min off the hot plate to cool down to room temperature.

### 2.4. Hardware Development

The probe and hardware block diagram are shown in Figure 1c. It consists of a microcontroller (component 1), a potentiostat (component 2), an SD card module (component 3), a battery (component 4), and the sensor (component 5). The probe measures 46 cm in length and is split into three sections. Section (i) houses the electronics in the top 5 cm. Section (ii) allows the adjustment of the depth of the sensors from 10 cm down to 40 cm, while section (iii) houses the sensor. A connfly cable connects the electronics at the top to the sensor location at the bottom of the probe. An Arduino MKR zero is used as a master to control the EmStat Pico and store the data on an SD card. The EmStat Pico derives its power from the MKR zero. The MKR zero was chosen as it comes equipped with an SD card module, thus negating the requirement of adding a separate SD card module and simplifying the programming complexity. The MKR zero has low operational and hibernation power consumption, making it perfect for portable solutions. Once the battery is connected, the MKR zero runs a preloaded code that sends a script to the EmStat Pico. The Pico runs SWV and then returns the voltage and current data back to the MKR zero, which are saved on the SD card. The SD card module is only powered up when saving the data and immediately powered down to prolong the battery life. Once the measurement is complete, the MKR zero sends a script that places the Pico in hibernation. The MKR zero then hibernates and wakes up after the preset time has passed to repeat the process.

### 2.5. Experimental Design

A printed circuit board (PCB) was designed to host the EmStat Pico and provide connectivity to a computer through a USB cable with a connector to plug the sensor in, as shown in Figure 1. All measurements were conducted through the PSTrace software version 8.5 provided by PalmSens. The prepared soil samples were incubated on the sensors for 10 min before any measurements were taken. For cyclic voltammetry measurements, the voltage was swept from −0.5 V to 0.7 V and the scan rate was set to 50 mV/s. For the squarewave voltammetry (SWV) experiments, the voltage was swept from 0 V to 0.7 V with a 50 mV step amplitude, a step size of 2 mV, and a frequency of 10 Hz. Two cycles of SWV with a 10 s grace period between them were run each time and the peak potential of the second scan was used for the calibration model. The first SWV scan usually has more than one peak where one is not dependent on pH [20]. Lastly, for the electro-impedance spectroscopy, the frequency was swept from 50 kHz to 5 Hz with a 10 mV AC signal and a 0 V DC bias. All plotting and statistical analyses were conducted using the statistical and graphing software Graph Pad Prism version 9.4 (Graph Pad Software Inc., La Jolla, CA, USA). The computational study was performed using Gaussian 16W software, Connecticut, USA, whereas the Gauss View 6 software, Connecticut, USA, was used to visualize the results and drawing of molecules. The optimization of structures such as alizarin and alizarin–H_2_O was conducted using the semi-empirical AM1 method first and then re-optimized with the Hartree–Fock method with a 6–31G (d) basis set. The results obtained from Hartree–Fock 6–31G (d) were considered for understanding the interaction and are presented herewith.

## 3. Results and Discussion

### 3.1. Computational Study of Alizarin and Its Interaction with Water Molecules

**Figure 3:**
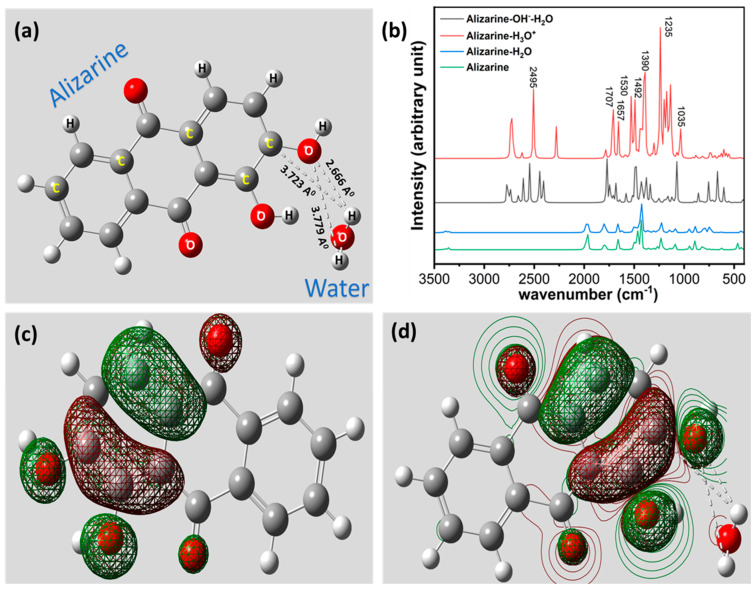
(**a**) Computational analysis of alizarin–water interaction shows strong H-bonded interaction. (**b**) Theoretical FTIR absorption spectra of alizarine with protonated and hydroxylated water molecule to justify the effect of pH. (**c**) HOMO electron cloud representation of alizarin. (**d**) HOMO–LUMO combined orbital configuration of alizarin, upon interaction with water molecule, shows significant tilt of electron cloud towards water molecule.

A computational study was performed to visualize the molecular structure of alizarin and its interaction with water molecules to understand the effect of pH at the molecular level. Water was chosen as a prime source of H^+^ for this computational study as it is the most abundant species present in soil contributing to the variation of soil pH. For this purpose, the structure of the alizarin–water interaction was optimized using Hartree–Fock, having a basis set of 6–31G (d). The optimized structure of the alizarin–water interaction is depicted in Figure 3a. The results depict a strong H-bonded interaction between O1 of the alizarin molecule and H1 of a water molecule. Moreover, two elongated H-bonded interactions are also found between O1 and C1 of alizarin and H1 and H2 of the water molecule, respectively.

To understand the effect of pH on the alizarin–water molecule, we visualized the interaction of the alizarin molecule with H_3_O^+^ and OH^−^-H_2_O, representing two different pH states of water: low and high, respectively. The interaction between H_3_O^+^ and alizarin is depicted in Figure S1, showing two distinct interactions with the alizarin molecule and the protonated water molecule with bond lengths of 2.121 A^0^ (O-H) and 4.409 A^0^ (H-H), respectively. The optimized structure of alizarin and OH^−^-H_2_O is depicted in Figure S2, showing three distinct interactions with bond lengths of 2.048 A^0^ (O-H), 1.226 A^0^ (H-H), and 1.064 A^0^ (O-H), respectively. The results show the absence of H-bonded interactions in a protonated and hydroxylated water molecule, whereas the presence of the same in neutral H_2_O with bond length 2.666 A^0^ is relatively closer to the H-bond length (2.7–3.3 A^0^). The results depict that the O-H bond length becomes shorter and even tends towards a covalent nature when protonation and hydroxylation increase, supporting the oxidation principle of alizarin.

We calculated the fundamental frequencies from the computational calculation and plotted them herewith. The fundamental FTIR plots of alizarin and alizarin–water are depicted in Figure 3b. The results show the presence of almost all the finger peaks of alizarin except one sharp peak at 2495 cm^−1^, duly present in protonated and hydroxylated water interacting with alizarin due to the OH-bonding interaction is shifted due to the effect of pH. The results also correlate with the experimental FTIR study, furnishing a stronger claim for the alizarin–water interaction. We also computed the difference of highest occupied molecular orbital (HOMO)–lowest unoccupied molecular orbital (LUMO) energy of the pristine alizarin molecule and alizarin–water complex to understand the stability of the complex compared to pristine alizarin. The graphical illustration of HOMO orbitals of alizarin is depicted in Figure 3c, showing that electron density populates at the Ƴ-aromatic ring, which is obvious. The inclusion of a single water molecule draws the HOMO electron cloud towards its vicinity and the HOMO orbitals of the alizarin–water complex can be seen at the α-aromatic ring closer to the water molecule, suggesting a strong affinity of alizarin towards the water molecule. The pictorial illustration of the same is depicted in Figure 3d. We have demonstrated the HOMO–LUMO value of alizarin and the alizarin–water complex in a table, depicted in Table 1.

We calculated the HOMO–LUMO energy gap of alizarin and water using Equations (1) and (2). From the calculation, it was found that the HOMO–LUMO energy gap was substantially reduced (~3X) after the formation of the alizarin–water complex. This suggests that the interaction is feasible. We also extracted the thermodynamics data, which show that the electronic + thermal Gibbs free energy of the alizarin–water complex is −910.11 Hartree, whereas the electronic + thermal Gibbs free energy of alizarin is −834.11 Hartree, which suggests that the formation of alizarin–water is indeed thermodynamically feasible too.

(1) $$\begin{array}{c} (E_{HOMO}^{Alizarin}-E_{LUMO}^{water})\;-\;(E_{LUMO}^{Alizarin}-E_{HOMO}^{water})=\\ (-0.31812\;-\;0.14940)\;-\;(+0.04172\;-\;(-)0.43743)=-0.46752\;-\;0.47915\\ =-0.94667\;Hartree \end{array}$$

(2) $$\begin{array}{c} (E_{HOMO}^{Alizarine–water}\;-\;E_{LUMO}^{Alizarine–water})=\\ -0.31002\;-\;(+)0.04603=-0.35605\;Hartree \end{array}$$

### 3.2. Electrochemical Characterization

**Figure 4:**
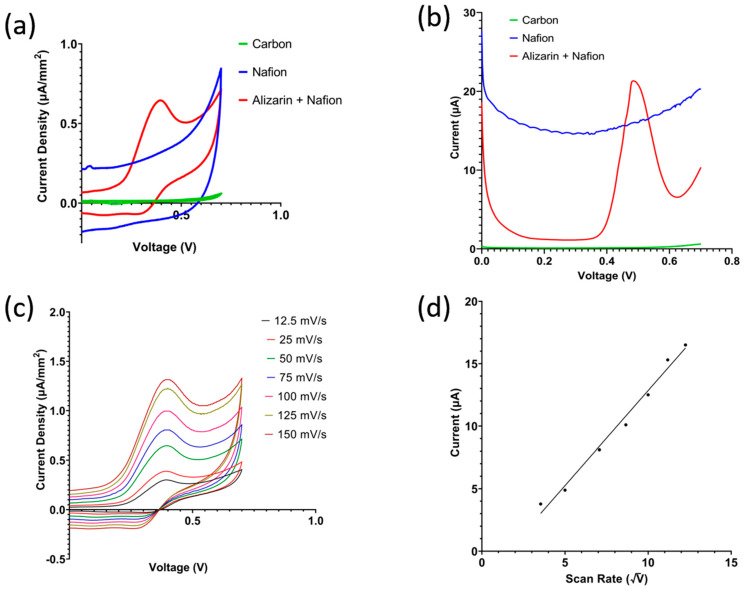
(**a**) Cyclic voltammetry showing oxidation peak due to presence of alizarin. (**b**) Effect of scan rate on the current density. (**c**) Peak current as calculated from arrow indication in (**b**) against square root of scan rate. (**d**) Squarewave voltammetry response of the bare electrode, modified by Nafion only, and modified with Nafion + alizarin mixture.

The effect of each layer in the sensor stack was studied by running cyclic voltammetry on three different sensors: a bare carbon sensor, a carbon sensor coated with 0.5% Nafion diluted in deionized water, and a carbon sensor coated with the Nafion and alizarin mixture. The sensors were coated with 10 mg of sandy loam soil slurry at pH 7 and incubated for 10 min. Cyclic voltammetry (CV) and squarewave voltammetry (SWV) demonstrate that the oxidation peak is due to the alizarin only, shown in Figure 4a,b, respectively. Figure 4c shows cyclic voltammetry recorded with varying scan rates ranging from 12.5 mV/s to 150 mV/s using the proposed sensor with the full Nafion and alizarin coating. Figure 4d shows the peak current measured from the baseline indicated in Figure 4c plotted against the square root of the scan rate. The presented linear relationship is in accordance with the Randles–Sevcik Equation (3), which depicts diffusion-controlled mass transfer [26].

(3) $$I_{p}=(2.69*10^{5})n^{\frac{3}{2}}{AD}^{\frac{1}{2}}{Cv}^{\frac{1}{2}}$$

I_p_ is the peak current from baseline, n is the stoichiometric number of electrons, A is the area of the working electrode, D is the diffusion coefficient of the electro-active species, C is the bulk concentration of electro-active species, and v is the scan rate. The redox peaks present in cyclic voltammetry are due to the oxidation of alizarin following Equation (4) where A is the alizarin, B is the anthracene-1,2,9,10-tetrone compound, and n is 2 as alizarin has two OH^−^ terminals.

(4) $$A↔B+nH^{+}+ne^{-}$$

### 3.3. Electrochemical Impedance Spectroscopy

**Figure 5:**
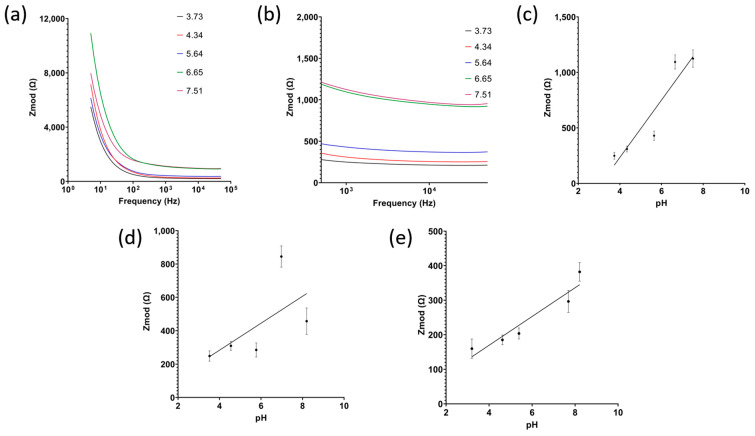
(**a**) Raw signals of electrochemical impedance spectroscopy with (**b**) zoomed in on 1 kHz range and (**c**) the calibrated dose response for clay soil, (**d**) sandy loam soil, and (**e**) loamy clay soil (*n* = 3, mean ± standard deviation).

The redox charge transfer process of alizarin in the presence of H^+^ ions was captured using electrochemical impedance spectroscopy (EIS). As pH increases, hydrogen ion concentration decreases, thus impedance increases. For each soil type, three different sensors were used to measure the impedance. The samples were incubated on the sensor for 10 min before the measurements. All plots and reported results show the mean and standard deviation. For clay soil, this behavior can be seen in Figure 5a–e, with the sensor having a sensitivity of 258.5 Ω/pH and R^2^ = 0.8686 for pH range 3–9. Similarly, loamy clay soil showed a linear relationship between impedance and pH, with a sensitivity of 30.5 Ω/pH and R^2^ = 0.8449.

However, when measuring the pH of sandy loam soil, it was observed that the impedance does not follow soil pH any longer. This can be attributed to the coarse particles of sandy soils. In clay and loamy soil, whose grains are fine and can always form a uniform layer on the electrode surface, the change in impedance measured is correlated with the charge transfer of H^+^ ions as the double layer size is consistent between samples. Sandy soils, on the other hand, have a coarse grain size with lots of spacing in between, creating an irregular double-layer formation at the electrode surface, thus the change in impedance measured is a combination of double-layer change and also the change in charge transfer.

### 3.4. Squarewave Voltammetry Study

**Figure 6:**
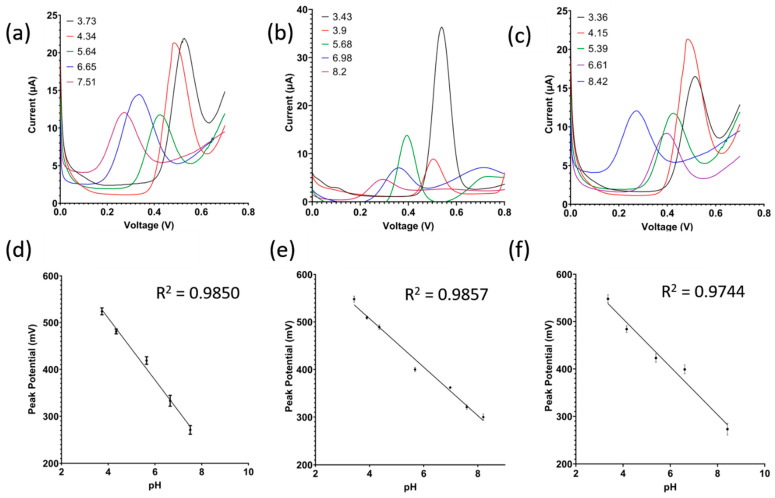
(**a**–**c**) Raw signals of squarewave voltammetry and (**d**–**f**) calibrated dose response for clay soil, sandy loam soil, and loamy clay soil, respectively (*n* = 3, mean ± standard deviation).

In the following study, squarewave voltammetry was used to measure the pH of the soil samples rather than EIS due to its higher sensitivity to faradaic current. Figure 4a,b confirm that the oxidation peak is due to the presence of alizarin in the coating and not produced by the carbon electrode or the Nafion matrix. The sharpness of the peak is attributed to the fact that squarewave voltammetry rejects capacitive current, and soil is capacitive in nature. Following that, the detection of pH in the three different soil types is shown in Figure 6a–c, showing a negative shift in the peak potential as pH increases, while the resultant linear model is shown in Figure 6d–f. The theoretical peak potential should vary by −59 mV/pH according to the Nernst equation at 25 C. As shown in Figure 6d–f, the sensor’s sensitivity varies between the different soils from −50 mV/pH up to −66 mV/pH, hovering close to the theoretical value of −59 mV/pH.

### 3.5. Validation

The efficacy of the system in providing an accurate pH level from the signal value was determined and the results are shown in Table 2. The pH of various soil samples of different textures was measured in deionized water using the reference glass electrode and compared to the pH measured using the proposed sensor. The samples measured by the reference electrode were prepared by mixing 5 g of soil with 5 mL of deionized water and stirred for 30 min, and then left to sit for another 30 min.

**Figure 7:**
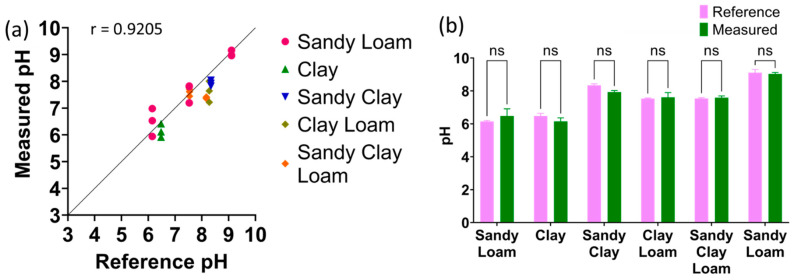
(**a**) Pearson’s correlation between measured pH using proposed sensor and glass electrode as the reference method. (**b**) The t-test results between the reference method and measured values (*n* = 3, mean ± standard deviation, ns: *p* > 0.05).

The samples measured by the sensors had their water content adjusted to 10–40% of the sample’s weight, mimicking agriculture conditions. All measurements were performed using three different sensors (*n* = 3). Figure 7a shows Pearson correlation analysis between all the measured pH values using the proposed sensor system plotted on the y-axis and the reference glass electrode pH values plotted on the x-axis, showing a Pearson r of 0.9205. Figure 7b shows the two-way ANOVA between the reference pH and measured pH for the various soil textures.

All samples had a *p*-value of higher than 0.05, indicating no significant difference between the reference pH glass electrode and the measured pH. These results show that creating three calibration curves of the textures at the ends of the soil texture triangle to cover all soil textures is viable. The measured averages, standard deviation, and calculated error percentages are reported in Table 2. The proposed sensor had an error rate of less than 10% across all samples, indicating that it is suitable for the in situ measurement of soil pH.

### 3.6. Real-Time Continuous In Situ Soil pH Monitoring

**Figure 8:**
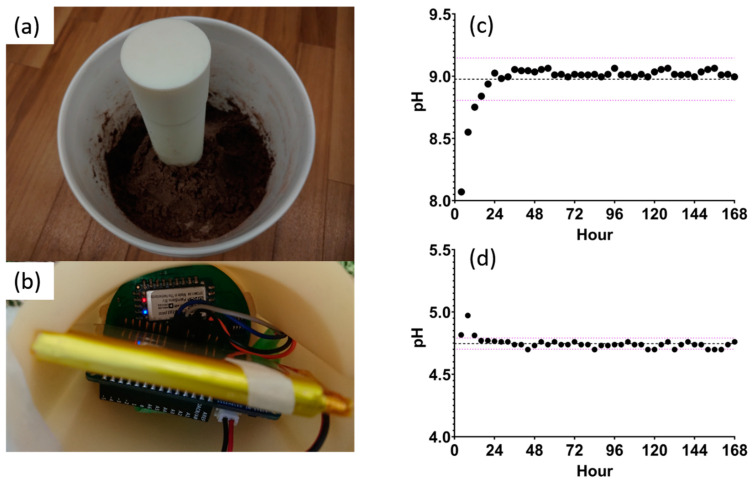
(**a**) Probe setup in the 19 L bucket and (**b**) the hardware housed at the top of the probe. (**c**) Measured pH over 7 days in sandy loam soil and (**d**) measured pH over 7 days in clay sandy soil with the mean and standard deviation lines drawn.

A temporal study over 7 days using SWV as the electrochemical method was set up. Figure 8a demonstrates the study setup. Two 19 L buckets were used to mimic an agriculture field. Bucket 1 contained sandy loam soil with a pH of 8.36, while bucket 2 had a mixture of 75% clay and 25% volcanic sand soil with a pH of 5.05. These soil types were chosen to test the sensor at the high and low ends of typical soil pH values. The moisture was maintained at 15–40% throughout the 7 days, mimicking an irrigation cycle in the field. The probe is inserted 20 cm into the soil so that the sensors are positioned 15 cm below the surface. The electronics are housed at the top of the probe, as shown in Figure 8b. A measurement was recorded every hour for 7 days. Every four data points were averaged and plotted against time, as shown in Figure 8c,d. It was observed that the sensor requires 12–24 h before stabilizing around the mean. Once a steady state is achieved, the measurements are within ±1 standard deviation from the mean, indicating excellent reliability. Table 3 summarizes the mean, standard deviation, and coefficient of variation for all data points. A coefficient of variance in buckets 1 and 2 of 1.885% and 0.945%, respectively, indicates high stability in the measured pH. To validate the results, the measured values are compared to the glass electrode as the reference method. For both soil types, the error rate is less than 10%, showing excellent accuracy and reliability.

## 4. Conclusions

An alizarin-based screen-printed electrode is presented for in situ soil pH sensing. The proposed sensor does not require any sample pre-treatment, thus accurately measuring the pH of unbuffered soil samples covering the pH range from 3 to 9. Squarewave voltammetry is the chosen electrochemical method of choice as it provides a pH-dependent response across the desired range, irrespective of the soil type. Three calibration curves were built to cover all soil types in the soil texture triangle. Sensor performance was validated against the gold standard glass electrode with less than a 10% error rate across the different soil types.

## Figures and Tables

**Table 1:** Calculated HOMO–LUMO energy of the alizarin, water, and alizarin–water.

Compound	E_HOMO_ (Hartree)	E_LUMO_ (Hartree)
Alizarin	−0.31812	+0.04172
Water	−0.43743	+0.14940
Alizarin–water	−0.31002	+0.04603

**Table 2:** Sensor validation results using squarewave voltammetry.

**Sample #**	**Soil Type**	**Calibration Curve**	**Reference pH (01.12.22)**	**Measured pH (06.09.22)**	**Std. Dev.**	**Error (%)**
1S1	Sandy Loam	Sandy Loam	6.15	6.48	0.43	5.43
1S2	Clay Loam	Clay	8.28	7.62	0.32	7.94
1S4	Sandy Clay Loam	Clay	7.54	7.59	0.11	0.63
1S3	Sandy Clay Loam	Clay	8.17	7.39	0.02	9.60
**Sample #**	**Soil Type**	**Calibration Curve**	**Reference pH (03.19.22)**	**Measured pH (06.09.22)**	**Std. Dev.**	**Error (%)**
2S2	Clay	Clay	6.48	6.15	0.21	5.11
2S1	Sandy Clay	Sandy Loam	8.34	7.93	0.09	4.94
2S4	Sandy Loam	Sandy Loam	7.53	7.61	0.29	1.03

**Table 3:** Statistics and validation data for both temporal studies.

**Descriptive Statistics**	**Bucket 1**		**Bucket 2**	
Number of values	42		42	
Mean	8.976		4.746	
Std. Deviation	0.169		0.045	
Std. Error of Mean	0.0261		0.0069	
Coefficient of variation	1.885%		0.945%	
**Validation**	**Measured**	**Reference**	**Measured**	**Reference**
Mean	8.976	8.36	4.746	5.05
Std. Deviation	0.169	0.08	0.045	0.03
Error rate	7.37%		6.02%	

## Data Availability

Data are contained within the article.

## Supplementary Materials

The following supporting information can be downloaded at: https://www.mdpi.com/article/10.3390/mi14122188/s1. Figure S1: Alizarine–H_3_O^+^ interaction is showing two distinct interactions between alizarine molecule and protonated water molecule. Figure S2: Alizarine-OH-H_2_O interaction is showing two distinct interactions between alizarine and hydroxylated water molecule.
