# Supplementary material for: Espial: Electrochemical Soil pH Sensor for In Situ Real-Time Monitoring
Source: Micromachines (Basel). 2023 Nov 30;14(12):2188. doi: 10.3390/mi14122188 (PMC10745296; doi:10.3390/mi14122188)
Supplement: Supplementary file 1 [file micromachines-14-02188-s001.zip › micromachines-2629234-supplementary.pdf]

Supplementary Information

Espial: Electrochemical soil pH sensor for in-situ real-time monitoring

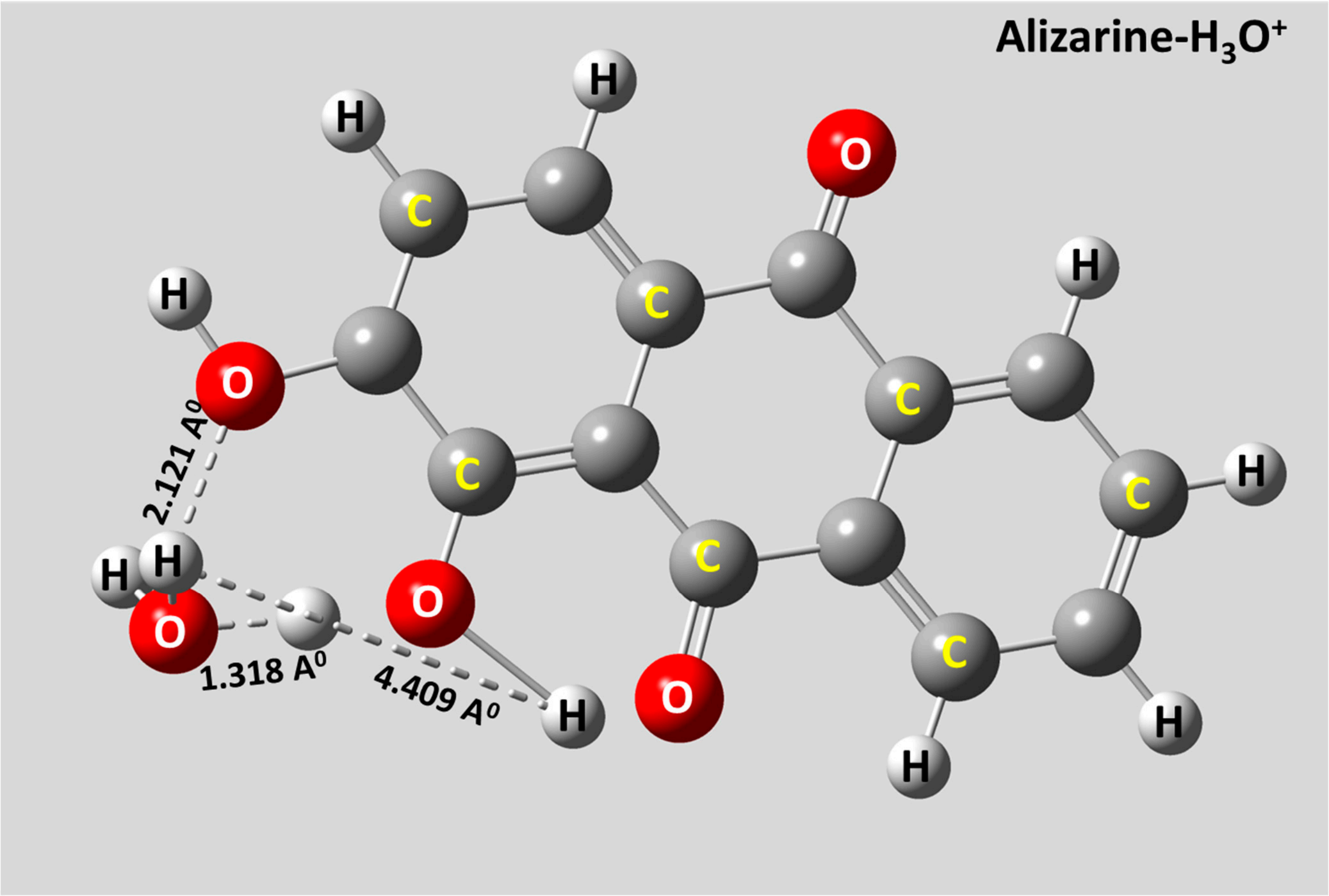

Figure S1. Alizarine–H$_3$O$^+$ interaction is showing two distinct interactions between alizarine molecule and protonated water molecule.

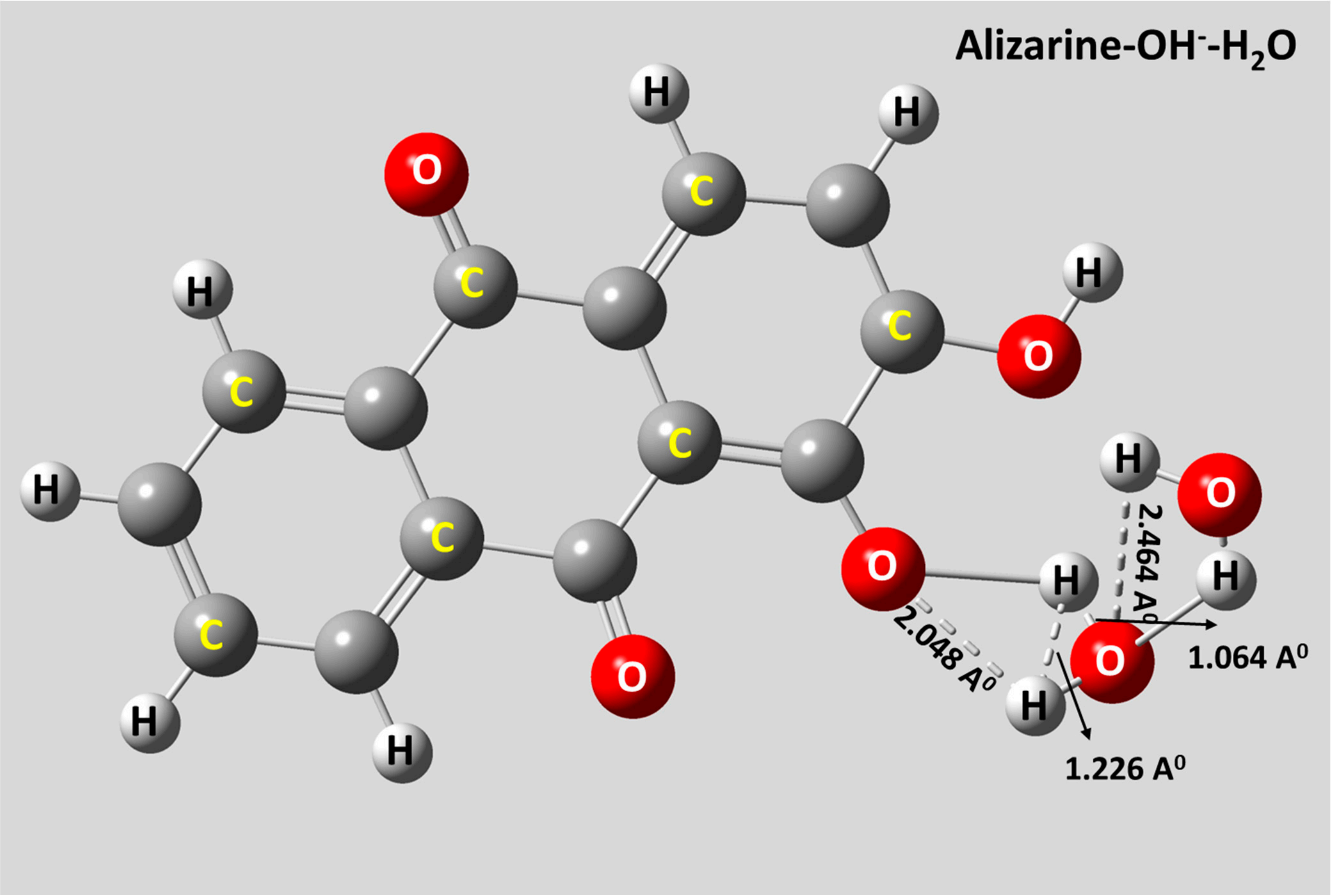

Figure S2. Alizarine-OH^{-}-H_2O interaction is showing two distinct interactions between alizarine and hydroxylated water molecule.
